# Robotic versus laparoscopic Gastrectomy for gastric cancer: a systematic review and updated meta-analysis

**DOI:** 10.1186/s12893-017-0290-2

**Published:** 2017-08-24

**Authors:** Ke Chen, Yu Pan, Bin Zhang, Hendi Maher, Xian-fa Wang, Xiu-jun Cai

**Affiliations:** 1 Department of General Surgery, Sir Run Run Shaw Hospital, School of Medicine, Zhejiang University, 3 East Qingchun Road, Hangzhou, Zhejiang Province 310016 China; 2 School of Medicine, Zhejiang University, 866 Yuhangtang Road, Hangzhou, Zhejiang Province 310058 China

**Keywords:** Laparoscopy, Robot, Gastrectomy, Stomach neoplasms, Morbidity, Meta-analysis

## Abstract

**Background:**

Advanced minimally invasive techniques including robotic surgery are being employed with increasing frequency around the world, primarily in order to improve the surgical outcomes of laparoscopic gastrectomy (LG). We conducted a systematic review and meta-analysis to evaluate the feasibility, safety and efficacy of robotic gastrectomy (RG).

**Methods:**

Studies, which compared surgical outcomes between LG and RG, were retrieved from medical databases before May 2017. Outcomes of interest were estimated as weighted mean difference (WMD) or risk ratio (RR) using the random-effects model. The software Review Manage version 5.1 was used for all calculations.

**Results:**

Nineteen comparative studies with 5953 patients were included in this analysis. Compared with LG, RG was associated with longer operation time (WMD = −49.05 min; 95% CI: -58.18 ~ −39.91, *P* < 0.01), less intraoperative blood loss (WMD = 24.38 ml; 95% CI: 12.32 ~ 36.43, *P* < 0.01), earlier time to oral intake (WMD = 0.23 days; 95% CI: 0.13 ~ 0.34, *P* < 0.01), and a higher expense (WMD = −3944.8 USD; 95% CI: -4943.5 ~ −2946.2, *P* < 0.01). There was no significant difference between RG and LG regarding time to flatus, hospitalization, morbidity, mortality, harvested lymph nodes, and cancer recurrence.

**Conclusions:**

RG can be performed as safely as LG. However, it will take more effort to decrease operation time and expense.

## Background

Laparoscopic gastrectomy (LG) has been widely used for the treatment of gastric cancer and a number of other different minimally invasive procedures have been developed to date [1, 2]. There are several benefits for patients; including better cosmesis, reduced pain, early recovery of intestinal function, and shorter hospital stay, while maintaining comparable oncologic safety [1–4].

Robotic surgery was first put into practice in 2000, after being approved by the US Food and Drug Administration (FDA). It plays an essential role in ergonomics and offers advantages such as motion scaling, less fatigue, tremor filtering, seven degrees of wrist-like motion, and three-dimensional vision [5, 6]. Surgeons hoped that such innovative technology could overcome some limitations innate to traditional laparoscopic surgery. Thus, experienced laparoscopic surgeons are increasingly trying to develop new procedures that best exploit the capabilities of robotic surgery in the treatment of gastric cancer [7].

Nonetheless, the present status of robotic gastrectomy (RG) is, as of the writing of this paper, still restricted and this is in part to due to the lack of randomized controlled trials (RCTs). Several previous studies including meta-analyses have argued that RG can be a more effective and safer operation in comparison with conventional LG. In spite of these studies, many questions still need to be answered, most notably, RG’s efficacy with regard to oncologic, long-term survival outcomes and its cost-effectiveness. Moreover, a series of studies on RG for the treatments of gastric cancer have been recently published. These studies are meaningful in highlighting the status of RG in the treatment of gastric cancer. Therefore, this paper’s current research is intended to conduct a comprehensive systematic review of all the currently available literature and a meta-analysis of RG in comparison to LG in order to assess the feasibility, security and efficacy of RG.

## Methods

### Search strategy

A systematic search of Web of Science, Cochrane Library, Embase, and PubMed was conducted to find studies comparing RG and LG for gastric cancer treatment published up until May 2017. Search terms included “gastric carcinoma”, “gastric cancer”, “laparoscopic”, “robotic”, and “gastrectomy”. The links in search results and references were also reviewed to find the additional literature. Based on the language competencies of the reviewers, English and Chinese were the only languages of searched papers.

### Eligibility criteria

The standards for research were comparative, using peer-reviewed studies of RG versus LG in gastric cancer for which the full texts were available. The most recent study or the study with the most subjects was chosen if overlapping research studies were found. Articles including any of the following were excluded: (1) Non-comparative studies such as letters, reviews, comments, posters, protocols, et al. (2) Studies including non-gastric carcinoma cases such as gastrointestinal stromal tumors, or benign gastric diseases; (3) Studies in which less than 2 of the interesting indices were reported.

### Data extraction and quality assessment

Two reviewers (Chen K and Pan Y) reviewed the publications thoroughly and independently. Data extracted included the following items: author, region, operation time, intraoperative estimated blood loss (EBL), time to flatus, time to oral intake, length of hospital stay (LOS), morbidity, mortality, costs, retrieved lymph nodes (RLN), proximal and distal margin distance, and long-term oncologic outcomes. In accordance with the morbidity reporting system of Memorial Sloan-Kettering Cancer Center [8], postoperative complications were categorized into medical complications (respiratory, cardiovascular, metabolic events, deep venous thrombosis, phlebitis, et al.) or surgical complications (bleeding, any complication required reoperation, anastomotic leakage or stricture, delayed gastric emptying, et al.). The means and standard deviations (SDs) were estimated as described by Hozo et al. [9] if the research offered medians and ranges. The choice of the articles included in this review adhered to the Preferred Reporting Items for Systematic Reviews and Meta-Analyses statement (PRISMA). The Newcastle-Ottawa Quality Assessment Scale (NOS) was utilized to evaluate the research quality (http://www.ohri.ca/programs/clinical_epidemiology/oxford.asp). The scale ranges from 0 to 9 stars: research with a score higher than or equal to 6 could be deemed as methodologically sound.

### Subgroup analysis

The uneven distribution of the surgical extension between the groups could affect the outcomes. Therefore, to eliminate the bias, a subgroup analysis of total or distal gastrectomy was conducted. It has been reported that robotic surgery may benefit obese patients, because of improved visualization, instrumentation, and ergonomics [10]. Therefore, we conducted a subgroup analysis to analyze the impact of operation-related factors on body mass index (BMI).

### Statistical analysis

The risk ratio (RR) was utilized to analyze the dichotomous variables and the weighted mean difference (WMD) was utilized to assess the continuous variables. Based on DerSimonian and Laird’s approach, the random-effects model was utilized to account for clinical heterogeneity, which refers to diversity in a sense that is relevant for clinical situations. According to the overall complication, the potential publication bias was determined by carrying out an informal visual inspection of funnel plots. The software of Review Manager version 5.1 (RevMan 5.1) was used to conduct data analysis. *P* < 0.05 was considered statistically significant.

## Results

### Studies selected

**Fig. 1:**
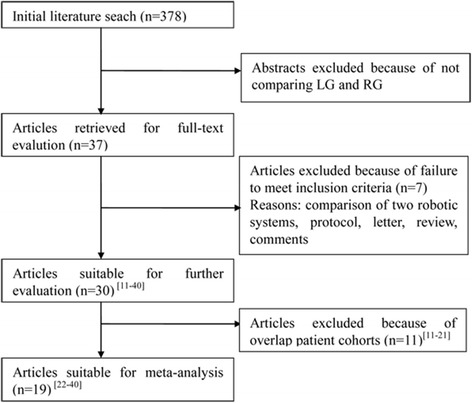
Flow chart of literature search strategies

A total of 378 potential articles, which were published from 1996 to 2017, were found. 37 articles were chosen based on the titles and abstracts, and then a thorough check of each text was conducted. Seven of them failed to meet our standards and were excluded. A further eleven papers were excluded due to overlapping patient cohorts (one from Hospital Niguarda Ca Granda, Italy [11]; one from Fujita Health University, Japan [12]; one from Taipei Veterans General Hospital, Taiwan [13]; four from Yonsei University, Korea [14–17]; four from National Cancer Center, Korea [18–21]). Finally, a total of nineteen studies were included for final meta-analysis [22–40]. A flow chart of the search strategies, which contains reasons for the exclusion of studies, is elucidated in Fig. 1.

### Study characteristics and quality

A total of 5953 patients were included in the analysis with 4123 undergoing LG (69.3%) and 1830 undergoing RG (30.7%). Most of the studies came from East Asia (10 Korea, 2 Japan, 4 China, 1 Taiwan) and 2 research studies came from Italy. The baseline features of the included studies are shown in Table 1; the evaluation of quality according to the NOS is shown in Table 2. NOS shows that four out of the 19 studies observed had 9 stars, one had 8 stars, seven had 7 stars and the remaining seven had 6 stars.

**Table 1:** Summary of studies included in the meta-analysis

Author	Region	Study design	Year	Study period	Sample size		Level of lymphadenectomy	Surgical extension	Reconstruction	Conversion (%)	
					LG	RG				LG	RG
Pugliese	Italy	OCS (R)	2010	2000–2009	48	16	D2	D	R-Y	3(6)	2(12)
Kim MC	Korea	OCS (P)	2010	2007–2008	11	16	D1 + β, D2	D	B-I, B-II	0	0
Kim KM	Korea	OCS (P)	2012	2005–2010	861	436	D1 + α/β, D2	D, T	B-I, B-II, R-Y	NR	NR
Son SY	Korea	OCS (R)	2012	2007–2011	42	21	D1 + β, D2	D, P, T	B-I, B-II, R-Y	NR	NR
Kang	Korea	OCS (P)	2012	2008–2011	282	100	D1 + α/β, D2	D, T	B-I, B-II, R-Y	E	0
Zhang	China	OCS (R)	2012	2009–2011	70	97	D2	D, P, T	B-I, B-II, R-Y	0	0
Hyun	Korea	OCS (P)	2013	2009–2010	83	38	D1 + α/β, D2	D, T	B-I, B-II, R-Y	0	0
Son T	Korea	OCS (R)	2014	2003–2010	58	51	D2	T	R-Y	0	0
Noshiro	Japan	OCS (P)	2014	2010–2012	160	21	D1 + α/β, D2	D	B-I, B-II, R-Y	0	0
Huang	Taiwan	OCS (P)	2014	2008–2014	73	72	D1 + α/β, D2	D, T	B-I, R-Y	NR	NR
Zhou	China	OCS (R)	2014	2010–2013	394	120	D1 + α/β, D2	D, P, T	B-I, B-II, R-Y	E	E
Liu	China	OCS (R)	2014	2012–2013	100	100	D2	D, P, T	B-I, B-II, R-Y	1(1)	0
Lee	Korea	OCS (P)	2015	2003–2010	267	133	D2	D	B-I, B-II, R-Y	NR	NR
Han	Korea	OCS (R)	2015	2008–2013	68	68	D1 + β	PPG	GG	0	0
Park	Korea	OCS (P)	2015	2009–2011	612	145	D1 + α/β	D, T	B-I, B-II, R-Y	10(1.6)	3(2.0)
Suda	Japan	OCS (R)	2015	2009–2012	438	88	D1 + α/β, D2	D, T	B-I, B-II, R-Y	0	0
Kim HI	Korea	OCS (P)	2016	2011–2012	185	185	D1 + α/β, D2	D, T	B-I, B-II, R-Y	2(1.1)	1(0.5)
Shen	China	OCS (R)	2016	2011–2014	330	93	D1 + α/β, D2	D, T	B-I, B-II, R-Y	0	0
Cianchi	Italy	OCS (R)	2016	2008–2015	41	30	D1 + α/β, D2	D	B-II, R-Y	0	0

*OCS* observational clinical study, *P* prospectively collected data, *R* retrospectively collected data, *D* distal gastrectomy, *P* proximal gastrectomy, *T* total gastrectomy, *PPG* pylorus-preserving gastrectomy, *B-I* Billroth-I, *B-II* Billroth-II, *R-Y* Roux-en-Y, *GG* gastro-gastro anastomosis, *E* exclude, *NR* not reported

**Table 2:** Quality assessment based on the NOS for observational studies

Author	Selection (Out of 4)				Comparability (Out of 2)	Outcomes (Out of 3)			Total (Out of 9)
	**①**	**②**	**③**	**④**		**⑤**	**⑥**	**⑦**	
Pugliese	*	*	*	*	**	*	*	*	9
Kim MC	*	*	*	*	*	*			6
Kim KM	*	*	*	*	*	*			6
Son SY	*	*	*	*	**	*			7
Kang	*	*	*	*	*	*			6
Zhang	*	*	*	*	**	*			7
Hyun	*	*	*	*	*	*			6
Son T	*	*	*	*	**	*	*	*	9
Noshiro	*	*	*	*	**	*			7
Huang	*	*	*	*	**	*			7
Zhou	*	*	*	*	**	*	*	*	9
Liu	*	*	*	*	**	*			7
Lee	*	*	*	*	*	*	*	*	8
Han	*	*	*	*	**	*	*	*	9
Park	*	*	*	*	*	*			6
Suda	*	*	*	*	*	*		*	7
Kim HI	*	*	*	*	*	*			6
Shen	*	*	*	*	**	*			7
Cianchi	*	*	*	*	*	*			6

①representativeness of exposed cohort

②selection of nonexposed cohort

③ascertainment of exposure

④outcome not present at the start of the study

⑤assessment of outcomes

⑥length of follow-up

⑦adequacy of follow-up

### Intraoperative effects and postoperative recovery

**Fig. 2:**
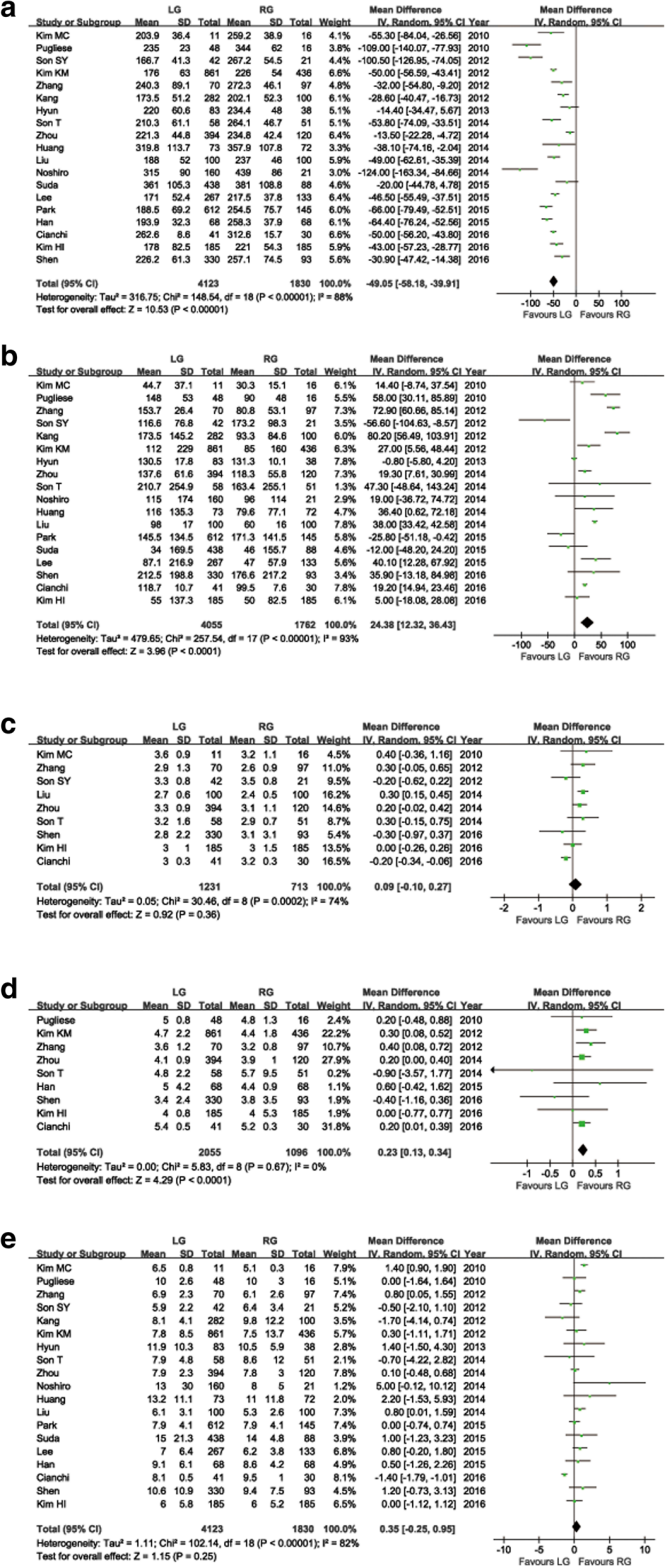
Forest plot of the meta-analysis for intraoperative effects and postoperative recovery. **a** Operation time. **b** Estimated blood loss. **c** Time to first flatus. **d** Time to restart oral intake. **e** Length of postoperative hospital stay

As shown in Table 1, three studies did not report the information of conversion; two studies excluded the conversion cases, whereas another nine research studies had no conversion. The pooled data based on four studies, which reported conversion cases, showed similar conversion rates between groups (RR = 0.88, 95% CI: 0.36 ~ 2.17, *P* = 0.78). A longer operation time for RG than for LG was reported in the majority of research and meta-analysis revealed that the average operation time of LG was 49.05 min shorter than RG (WMD = −49.05 min; 95% CI: -58.18 ~ −39.91, *P* < 0.01) (Fig. 2a). Intraoperative EBL was reported in eighteen of the research studies, which was lower in RG than LG (WMD = 24.38 ml; 95% CI: 12.32 ~ 36.43, *P* < 0.01) (Fig. 2b).

The pooled mean time to first flatus indicated no significant difference between the two groups (WMD = 0.09 days; 95% CI: -0.10 ~ 0.27, *P* = 0.36) (Fig. 2c). Nonetheless, according to the meta-analysis, the mean time to restart oral intake was longer in LG than in RG (WMD = 0.23 days; 95% CI: 0.13 ~ 0.34, *P* < 0.01) (Fig. 2d). All studies reported the LOS. According to the pooled data, a significant difference did not exist between the two groups with regard to LOS (WMD = 0.35 days; 95% CI: -0.25 ~ 0.95, *P* = 0.25) (Fig. 2e). All intraoperative effects and postoperative recovery outcomes are summarized in Table 3.

**Table 3:** Results of the meta-analysis

Outcomes	No. of studies	Sample size		Heterogeneity (*P, I* ^*2*^)	Overall effect size	95% CI of overall effect	*P*
		LG	RG				
Conversion	4	16	6	0.68, 0%	RR =0.88	0.36 ~ 2.17	0.78
Operation time (min)	19	4123	1830	<0.001, 88%	WMD = −49.05	-58.18 ~ −39.91	<0.01
Blood loss (mL)	18	4055	1762	<0.001, 93%	WMD =24.38	12.32 ~ 36.43	<0.01
Time to first flatus (days)	9	1231	713	<0.001, 74%	WMD =0.09	-0.10 ~ 0.27	0.36
Time to oral intake (days)	9	2055	1096	0.67, 0%	WMD =0.23	0.13 ~ 0.34	<0.01
Hospital stay (days)	19	4123	1830	<0.001, 82%	WMD =0.35	-0.25 ~ 0.95	0.25
Overall complications	19	4123	1830	0.82, 0%	RR =0.96	0.82 ~ 1.13	0.65
Surgical complications	17	3234	1552	0.52, 0%	RR =0.87	0.72 ~ 1.05	0.15
Medical complications	12	2137	907	0.82, 0%	RR =1.34	0.75 ~ 2.40	0.32
Reoperation	7	1796	789	0.35, 11%	RR =0.69	0.29 ~ 1.62	0.39
Mortality	7	2131	838	0.91, 0%	RR =0.67	0.26 ~ 1.74	0.41
Retrieved lymph nodes	17	3229	1585	<0.001, 86%	WMD = −1.44	-3.26 ~ 0.37	0.12
Proximal margin (cm)	9	2006	1024	0.21, 26%	WMD = −0.14	-0.36 ~ 0.07	0.18
Distal margin (cm)	8	1948	973	<0.001, 81%	WMD =0.09	-0.46 ~ 0.65	0.74
Recurrence	3	500	187	0.39, 0%	RR =1.09	0.57 ~ 2.05	0.80
Cost (USD)	4	390	384	<0.001, 93%	WMD = −3944.8	-4943.5 ~ −2946.2	<0.01

### Morbidity and mortality

**Fig. 3:**
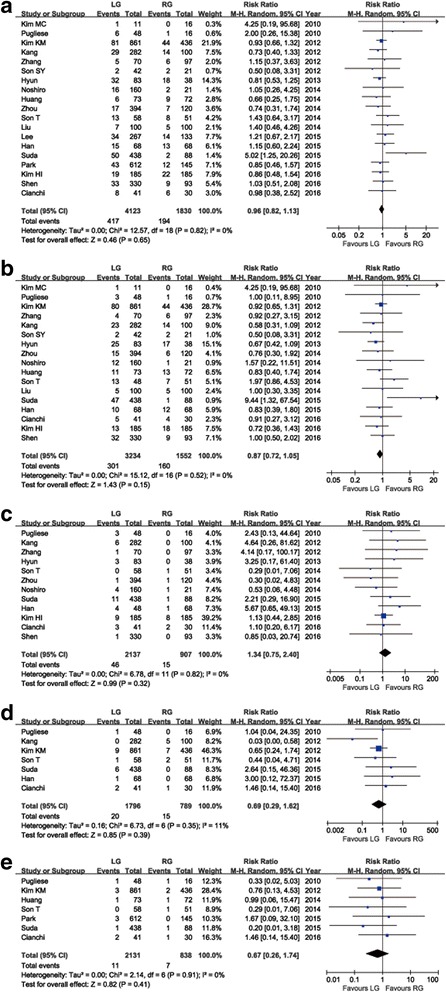
Forest plot of the meta-analysis for morbidity and mortality. **a** Overall postoperative complications. **b** Surgical complications. **c** Medical complications. **d** Reoperation. **e** Mortality

**Fig. 4:**
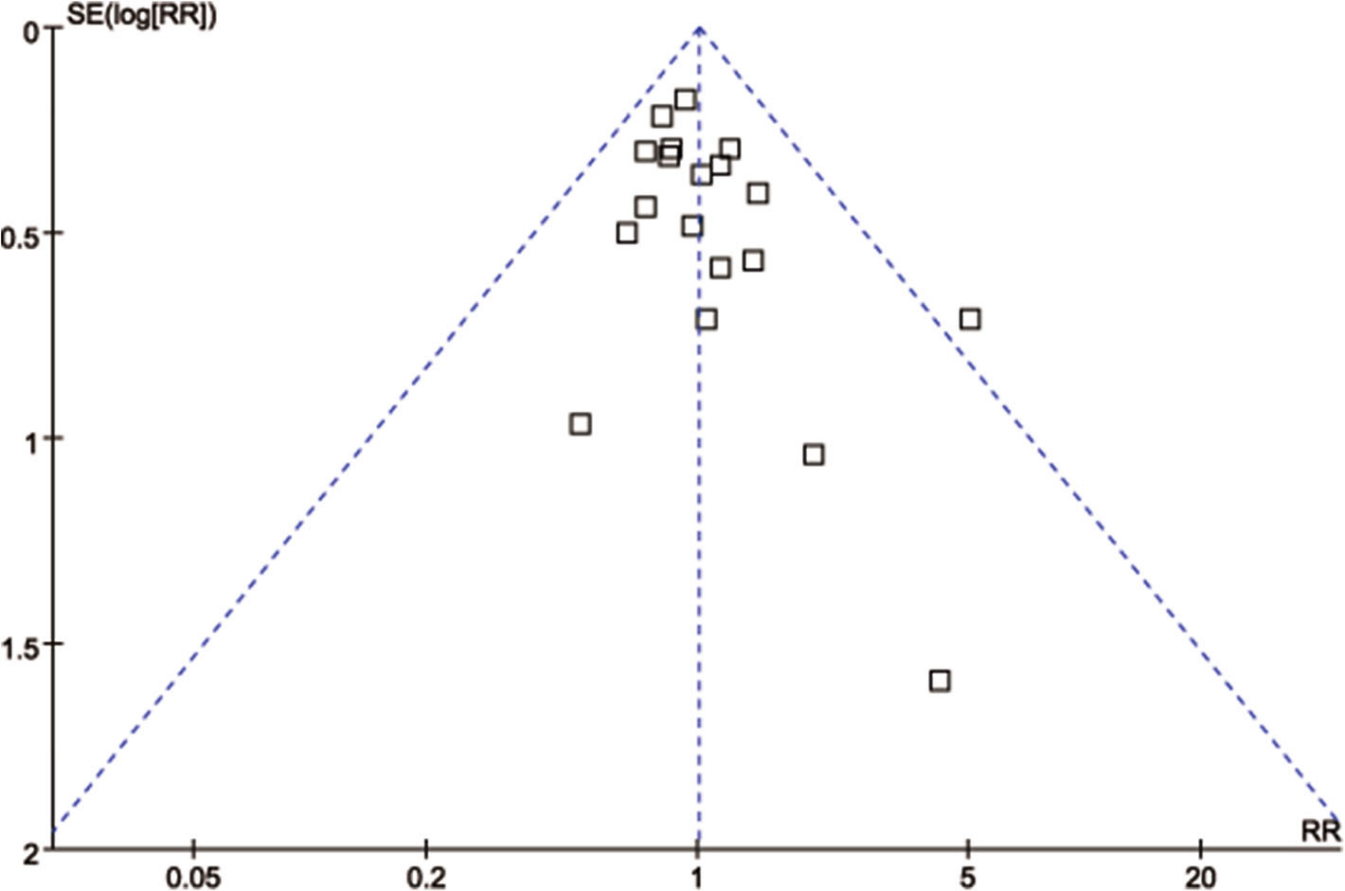
Funnel plot of the overall postoperative complications

All studies reported adverse incidents ranging from 0% to 47.4% in RG and from 4.3% to 38.6% in LG. No significant difference in the rate of overall postoperative complications was identified between the groups of RG and LG (RR = 0.96, 95% CI: 0.82 ~ 1.13, *P* = 0.65) (Fig. 3a). Symmetry was shown in the visual inspection of the funnel plot, showing no severe publication bias (Fig. 4). After further analysis, surgical complications were similar between groups (RR = 0.87, 95% CI: 0.72 ~ 1.05, *P* = 0.15) (Fig. 3b), as were the medical complications (RR = 1.34, 95% CI: 0.75 ~ 2.40, *P* = 0.32) (Fig. 3c). Reoperation cases were reported in seven studies, and there was no significant difference in the reoperation rates (RR = 0.69, 95% CI: 0.29 ~ 1.62, *P* = 0.39) (Fig. 3d). Also, seven studies reported mortality and no significant difference could be found in postoperative mortality (RR = 0.67, 95% CI: 0.26 ~ 1.74, *P* = 0.41) (Fig. 3e). The specific reoperation and causes of mortality reported in the studies are summarized in Table 4. The meta-analysis results on morbidity and mortality are outlined in Table 3.

**Table 4:** Systematic review of the specific reoperation and death reasons

Author	Group	Reoperation	Death
Pugliese	LG	Enterocutaneous leak (*n* = 1)	Severe bleeding due to hepatic failure (*n* = 1)
	RG	NC	Hemorrhagic stroke (*n* = 1)
Kim KM	LG	Leak-related (*n* = 4)^a^	Leak-related (*n* = 2)^a^
	RG	Leak-related (*n* = 6)^a^	NC
	RG	Leakage and obstruction (*n* = 5)	NC
Lee	LG	Anastomotic leakage (*n* = 1)	NC
	RG	Anastomotic leakage (*n* = 1), anastomotic bleeding (*n* = 1)	Anastomotic bleeding (*n* = 1)
Huang	LG	NC	Duodenal stump leakage (*n* = 1)
	RG	NC	Gastrojejunostomy leakage (*n* = 1)
Han	LG	Intra-abdominal bleeding due to liver capsular injury (*n* = 1)	NC
Park	LG	NC	Immediate postoperative bleeding (*n* = 1), mesenteric infarction (*n* = 1), septic shock caused by afferent loop syndrome (*n* = 1)
Cianchi	LG	NC	Duodenal stump leakage with peritonitis and sepsis (*n* = 1), acute myocardial infarction (*n* = 1)
	RG	Intestinal occlusion (*n* = 1)	Cerebral vascular accident (*n* = 1)

*NC* no case

^a^: included anastomotic leakage and duodenal stump leakage

### Oncologic outcomes and long-term survival

**Fig. 5:**
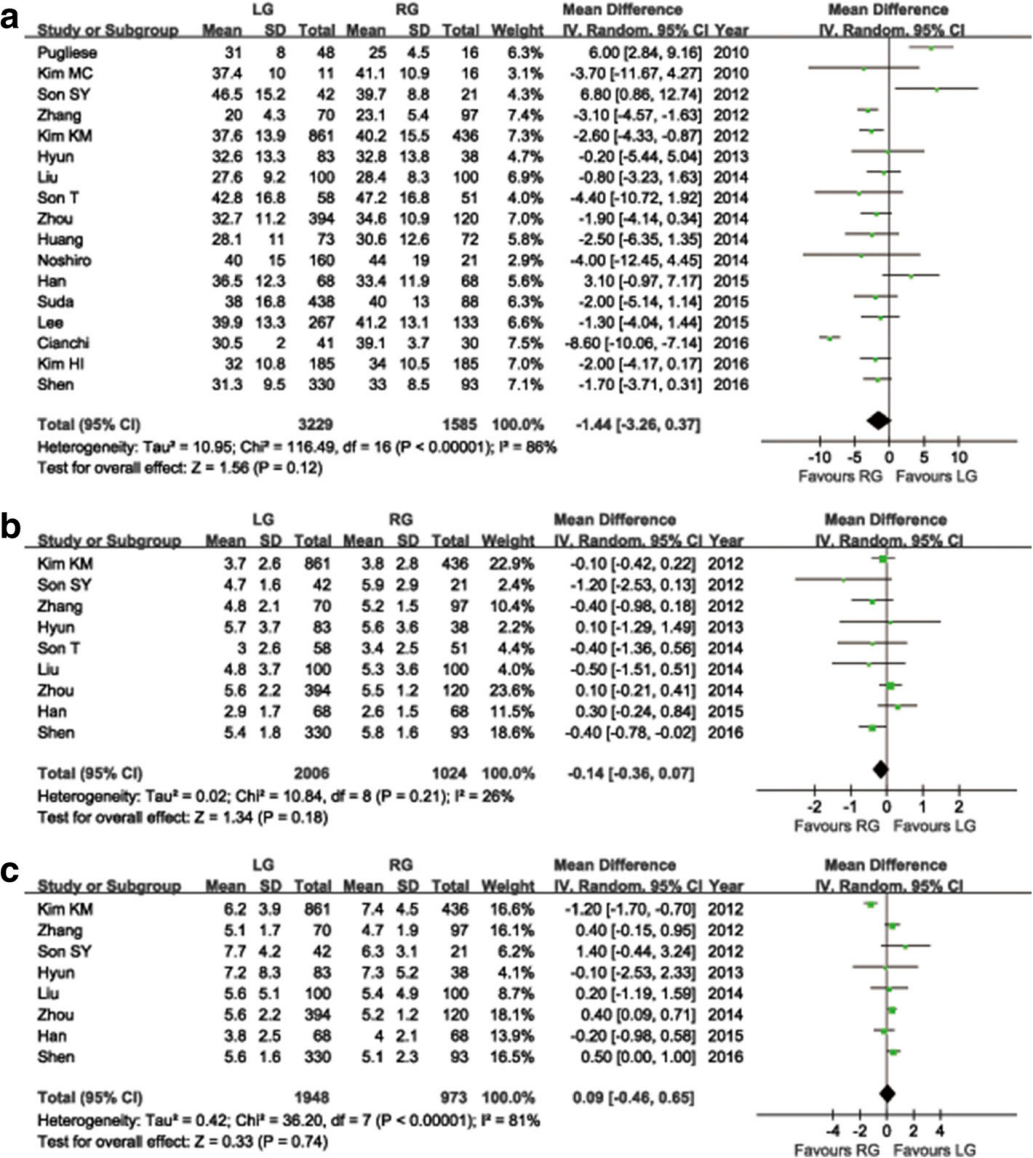
Forest plot of the meta-analysis for oncologic outcomes. **a** Number of retrieved lymph nodes. **b** Proximal margin distances. **c** Distal margin distance. **d** Cancer recurrence

The differences in the average number of RLNs were not considerable in the pooled statistics with a tendency towards a reduction in the LG group when compared to the RG group (WMD = −1.44; 95% CI: -3.26 ~ 0.37, *P* = 0.12) (Fig. 5a). The distal or proximal margin distances were described in nine studies. Meta-analysis of the proximal margin distances showed no significant difference between the two groups (WMD = −0.14 cm; 95% CI: -0.36 ~ 0.07, *P* = 0.18) (Fig. 5b), the same applies to the distal margin distance (WMD = 0.09 cm; 95% CI: -0.46 ~ 0.65, *P* = 0.74) (Fig. 5c). Cancer recurrence was reported in three research studies and the pooled data indicated that the difference between RG and LG was not significant (RR = 1.09, 95% CI: 0.57 ~ 2.05, *P* = 0.80). Long-term survival rates were reported in three research studies, and no considerable difference in the survival rates between the LG group and RG group could be found. In addition, during the follow-up time, no significant difference in the survival rates between both of the groups could be found in the studies of Lee et al. [34] and Han et al. [35] though they failed to report the particular survival rates. The meta-analysis of survival rates cannot be done due to the limited data. The systematic review outcomes of follow-up time, recurrence patterns and sites, and long-term survival rates are summarized in Table 5.

**Table 5:** Systematic Review of Recurrence and Long-term Survivals

Author	Group	Stage	Chemotherapy	Follow-up (mo)	Recurrence	Survival (%)
Pugliese	LG	Any TNM_0_	T_3_ or any TN_+_	53 (3–112)	8^a^	3y–OS: 85; 5y–OS: 83^&^
	RG			28 (2–44)	4^a^	3y–OS: 78^&^
Son T	LG	Any TNM_0_	NR	70	3^b^	5y–DFS: 91.2; 5y–OS: 91.1
	RG				3^b^	5y–DFS: 90.2; 5y–OS: 89.5
Zhou	LG	Any TNM_0_	Routinely^#^	17(3–41)	28	1, 2, 3-OS: 87.3, 77.1, 69.9 3y–OS N_−_:82.6, 3y–OS N_+_:60.3
	RG				5	1, 2, 3-OS: 90.2, 78.1, 67.8 3y–OS N_−_: 84.4, 3y–OS N_+_: 57.5
Lee	LG	Any TNM_0_	NR	75	NR	NSD
	RG					
Han	LG	cT_1-2_N_0_M_0_	3 cases (4.4%)^$^	19.3	0	NSD
	RG		3 cases (4.4%)^$^	22.7	0	

Follow-up time were shown as median (range) or median only

*DFS* disease-free survival, *OS* overall survival, *y* year, *N*_−_ negative nodal metastasis, *N*_*+*_ positive nodal metastasis, *NR* not report, *NSD* only reported no significant difference between two groups without specific survival rate

^a^some patients had mixed tumor recurrence, identified recurrence in LG: local (*n* = 2), peritoneum (*n* = 2), liver (*n* = 1), lung (*n* = 2), bone (*n* = 1); identified recurrence in RG: peritoneum (*n* = 1), liver (*n* = 1), bone (*n* = 1). &: for overall patients, 5y–OS N_−_: 97%, 5y–OS N_+_: 52%

^b^LG, peritoneum (*n* = 2), lung (*n* = 1); RG, breast (*n* = 1), splenic hilum (*n* = 1), ovary (*n* = 1). #: 5-fluorouracil + oxaliplatin intravenous chemotherapy. $: because of advanced disease status after surgery

### Total cost

**Fig. 6:**
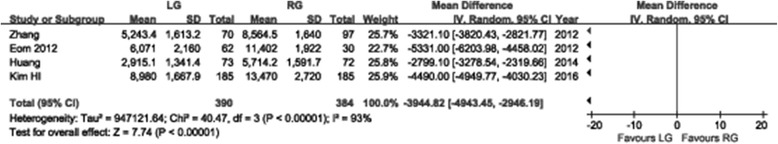
Forest plot of the meta-analysis for total cost

Only four studies recorded their total cost and they all reported a higher cost for RG than LG. The meta-analysis demonstrated that the total cost of RG groups was significantly higher than LG groups (WMD = −3944.8 USD; 95% CI: -4943.5 ~ −2946.2, *P* < 0.01) (Fig. 6).

### Subgroup analysis of distal or total gastrectomy

For the subgroup analysis of distal gastrectomy (DG), the RG group still holds the longer operation time (*P* < 0.01), lower EBL (*P* < 0.05) and with similar LOS, overall complications, mortality as well as RLN (*P* > 0.05). However, there was a reduced time to oral intake for RG, but with only a marginal difference compared to the LG group (*P* = 0.05). As for total gastrectomy (TG), there is no large difference between the outcomes of operation time, EBL, time to oral intake, LOS, overall complications and mortality against DG subgroup analysis, the number of RLNs of RG was more than that of LG with a significant difference (*P* = 0.03). The subgroup analysis results of surgical extension are summarized in Table 6. Generally speaking, the difference in surgical extension had little effect on the overall meta-analysis results.

**Table 6:** Results of the subgroup analysis of distal or total gastrectomy

Outcomes	No. of studies	Sample size		Heterogeneity (*P, I* ^*2*^)	Overall effect size	95% CI of overall effect	*P*
		LG	RG				
Operation time (min)							
DG	8	1635	453	<0.001, 78%	WMD = −57.08	−68.62 ~ −45.54	<0.01
TG	5	448	166	0.004, 74%	WMD = −42.62	−66.72 ~ −18.52	<0.01
Blood loss (mL)							
DG	8	1635	453	<0.001, 77%	WMD =19.27	3.86 ~ 34.68	0.01
TG	5	448	166	0.54, 0%	WMD =23.77	1.97 ~ 45.56	0.03
Time to oral intake (days)							
DG	3	344	116	0.49, 0%	WMD =0.18	0.00 ~ 0.36	0.05
TG	3	251	100	0.71, 0%	WMD = −0.18	−0.55 ~ 0.20	0.36
Hospital stay (days)							
DG	8	1635	453	<0.001, 92%	WMD =0.52	−0.69 ~ 1.74	0.40
TG	5	448	166	0.75, 0%	WMD =0.28	−0.80 ~ 1.36	0.61
Overall complications							
DG	8	1635	453	0.86, 0%	RR =1.19	0.83 ~ 1.71	0.34
TG	4	330	140	0.49, 0%	RR =1.32	0.80 ~ 2.18	0.27
Mortality							
DG	4	942	213	0.84, 0%	RR =0.84	0.21 ~ 3.30	0.80
TG	2	194	81	0.55, 0%	RR =0.15	0.02 ~ 1.41	0.10
Retrieved lymph nodes							
DG	8	1635	453	<0.001, 92%	WMD = −2.10	−5.90 ~ 1.70	0.28
TG	5	448	166	0.63, 0%	WMD = −2.51	−4.83 ~ −0.19	0.03

*DG* distal gastrectomy, *TG* total gastrectomy

### Subgroup analysis of weight influence

Only two studies had data for subgroup analysis based on weight [28, 34]. The patients were divided based on preoperative BMI into non-overweight (BMI < 25 kg/m^2^) and overweight (BMI >25 kg/m^2^) groups. In the non-overweight subgroup, the RG group still had a longer operation time (*P* < 0.01), while in the overweight subgroup; the operation time was similar between groups (*P* = 0.27). In addition, there was no significant difference between LG and RG for the outcomes of EBL and RLNs regardless of overweight or non-overweight subgroup. Other perioperative outcomes cannot be analyzed due to the limited data. The subgroup analysis results based on weight are summarized in Table 7.

**Table 7:** Results of the subgroup analysis of weight

Outcomes	No. of studies	Sample size		Heterogeneity (*P, I* ^*2*^)	Overall effect size	95% CI of overall effect	*P*
		LG	RG				
Operation time (min)							
non-overweight	2	232	127	0.06, 72%	WMD = −37.63	−62.82 ~ −12.43	<0.01
overweight	2	118	44	0.008, 86%	WMD = −28.58	−79.11 ~ 21.94	0.27
Blood loss (mL)							
non-overweight	2	232	127	0.11, 60%	WMD =0.90	−13.44 ~ 15.25	0.90
overweight	2	118	44	0.03, 80%	WMD =39.84	−41.71 ~ 121.39	0.34
Retrieved lymph nodes							
non-overweight	2	232	127	0.34, 0%	WMD = −1.88	−4.78 ~ 1.01	0.20
overweight	2	118	44	0.03, 79%	WMD =4.32	−4.10 ~ 12.74	0.31

## Discussion

The cost-effectiveness and definite advantages of RG have not been well documented, which is different when compared to the evolution of LG versus conventional open surgery [20]. However, the number of publications on RG has gradually increased in recent years. The oncologic outcomes, postoperative outcome, intraoperative effects and costs of a total of 1830 patients who underwent RG for gastric cancer treatment in 19 studies were reviewed as we believe such research would contribute to a more objective and comprehensive assessment of the current RG surgical status.

In spite of the considerable heterogeneity, the prolonged operating time in RG was shown in almost all the included research studies. The prolonged exposure time to pneumoperitoneum and the associated increased time of anesthesia is a major concern. Few publications describe the effect of longer operation times during RG. However, previous research of LG in senior patients has shown that longer operation time did not result in detrimental effects with regard to surgical results [41]. Therefore, a prolonged operating time should not affect surgeons directly on conducting research on RG’s new utility. Inevitably, the docking time was considered as an essential factor, which enhanced the operating time. The docking time was between 20 min to 60 min as reported in our study [7, 13, 15, 31], We found RG had longer operation times than LG by 49 min, which suggested the ‘true’ time spent on operations was similar or even shorter than LG. Furthermore, with the increased utilization of the new robotic surgical system, operation times are expected to shorten. Several studies have reported that the da Vinci Xi robotic platform is more user-friendly and is easier to install in rectal and nephritic surgery [42, 43]. As a result, we believed that RG is technically feasible with regard to operation time.

Surgeons have to go through a learning curve to master a technique. The surgical results, such as operation time, oncological outcomes and postoperative complications can be affected by surgeon’s familiarity with the instrument, experience and assistant compliance. In general, before stabilization, LG should be conducted on around 40 to 60 cases [44]. The learning curve for RG was shorter for experienced surgeon who had performed LG, which is forecasted to be only 10 to 20 cases [12, 13, 18, 26]. A surgeon experienced in laparoscopic surgery can conduct robotic surgery securely even in their first case [16]. Several studies investigated in this meta-analysis compared the initial and later experiences of robotic surgery [12, 13, 18, 26]. The later cases performed by the same surgical team could progress toward shortening operation times.

Postoperative morbidity is the main indicator for assessing the safety and feasibility of one procedure. It is widely accepted that laparoscopic surgery for gastric cancer is safer and could have fewer complications than open surgery [45]. Our meta-analysis demonstrated a comparable complication rate in RG versus LG group, and the low heterogeneity regardless of overall, surgical or medical complications encourages us to believe that RG indeed is as safe as LG. Improvements such as three-dimension images and tremor filtering could theoretically contribute to safer implementations of the robotic system for gastrectomy and lymphadenectomy. According to the multivariate analyses in the Suda study, the application of RG was an important independent protective factor in regards to the postoperative complication [37]. Tokunaga et al. [46, 47] reported the incidences of overall adverse events after RG which were 14.2% and 22.2% based on their two-phase II studies, which are comparable to the rates of 19–27% in previous studies of LG [48, 49].

Obesity is one of the most significant health problems today and rates are still increasing around the world. Some studies claim obesity causes increased blood loss, operation time, and wound infection rate et al. [50, 51], whereas others did not observe any negative effect on surgical outcomes [52]. Recently, Harr et al. [10] showed that the benefits of robotic methods were more evident in high versus normal BMI patients when performing a colostomy. The authors concluded that robotic surgery might overcome the difficulties associated with thick abdominal walls and excessive intra-abdominal fat, thanks to improved visualization, instrumentation, and ergonomics [10]. However, compared to other operations such as the colorectal or prostatic surgeries, which are in relatively narrow regions, the superiority of da Vinci over the laparoscopy may not be obvious, in that gastric surgery is conducted in the upper abdomen of a relatively spacious location. In our study, the overall mean operation time of RG and LG were similar in the overweight subgroups, contrasting with those in the non-overweight subgroups, which implied RG to be superior to LG when used on overweight patients. However, the sample size of the overweight subgroups was not large enough to be conclusive.

The traditional straight forceps in LG fail to enable surgeons to reach deep-seated vessels and other areas, like the supra pancreatic one, in which the dissection of lymph nodes around the splenic hilum, splenic artery, and hepatic artery areas is deemed extremely hard. The tremor filtering, wristed instruments, as well as stable exposure and high-solution image can help surgeons thoroughly retrieve the lymph nodes around the delicate areas [21]. According to one included study, the amount of RLNs was considerably higher with robotic surgery in the splenic hilum and splenic artery areas [29]. Our meta-analysis shows adequate RLNs with means of 35.4 and 36.1 in the LG and RG groups, respectively. The mean number of RLNs of RG was more than that of LG with a marginal difference observed in the pooled data, even though most studies had been done during initial implementation of the robotic technique. Therefore, we believe that robotic technique could be superior to the conventional laparoscopic technique for lymphadenectomy. Since the history of the clinical application of RG is a short one, few reports have compared long-term survival outcomes with other methods. Coratti et al. [53] demonstrated that the 5-year survival rate after RG stratified with Stage IA, IB, II, and III was 100%, 84.6%, 76.9%, and 21.5%, respectively. Pugliese et al. [11] reported a cumulative overall 5-year survival rate of 78% with a mean follow up of 30 months (range 2–86) after RG for gastric cancer.

The application of robotic surgery remains controversial, mainly due to the considerable expense. The total difference in cost between the LG and RG groups has been predicted to be around 3900 USD [18, 31, 38], which is mainly derived from the robotic system itself. According to the opinions of some investigators, the higher cost of robotic surgery is not enough to justify the theoretical advantages of this technology [54]. If RG can reduce complications and shorten hospital stay, the higher costs of the robotic system would be partially offset. Based on this, it is essential for robotic operators to inspect whether the potential advantages of the robotic approach justifies its high cost in the treatment of gastric cancer.

Our research has the following limitations: (1) Selection bias: As no RCT was available to be included in the meta-analysis due to the higher cost of robotic surgery, selection biases are inevitable in surgical abstention which should be carefully interpreted. (2) Clinical heterogeneity: The homogeneity test for the continuous variables exhibited substantial heterogeneity due to the inherent flaws of a retrospective study, the uneven surgical skills of the different surgeons, as well as regional differences, etc. More importantly, for surgeons in the East, radical distal gastrectomy for middle and distal gastric cancer is popular [55], while the distal subtotal is preferred in the West [56]. Thus we cataloged distal gastrectomy and subtotal gastrectomy as a subgroup. Though it brings some interesting results due to the expansion of sample size, such a combination would result in clinical heterogeneity. (3) Regional difference: The majority of the included studies came from East Asia, because East Asia has the highest prevalence of gastric cancer, while gastric cancer is relatively uncommon in Western countries. Besides, in East Asia, particularly Korea, Japan and some areas of China, the proportion of early gastric cancer has increased as a result of the improved surveillance of gastric cancer in these regions [57, 58]. On the other hand, although increasing evidence continues to show no difference between patients undergoing open or laparoscopic surgery for oncologic outcomes, the Japanese Gastric Cancer Association still classifies minimal invasive surgery as investigational treatment and only recommends minimal invasive surgery for early stage gastric cancer patients [55]. Therefore, the cases in our studies, especially those from East Asia, were mainly early stage cases. All of the above limitations must be kept in mind when interpreting the results of our study.

## Conclusions

Except for the longer operation time and higher costs, RG for the patients with gastric cancer was not inferior to LG. Besides; RG holds the potential benefits for larger numbers of lymph node dissection and reduced intraoperative blood loss. Further prospective studies are needed in order to confirm these advantages. In addition, long-term results are needed, particularly for the oncological adequacy of robotic gastric cancer resections.

## Abbreviations

BMI: body mass index
EBL: estimated blood loss
LG: laparocopic gastrectomy
LOS: length of hospital stay
NOS: Newcastle-Ottawa Quality Assessment Scale
RCT: randomized controlled trial
RG: robotic gastrectomy
RLN: retrieved lymph nodes
RR: risk ratio
SD: standard deviation
WMD: weighted mean difference
